# Synthesis and polymerization of 1-(2-diallylaminoethyl)pyrimidines

**DOI:** 10.1080/15685551.2018.1448232

**Published:** 2018-03-23

**Authors:** Jomana Elaridi, Alaa Ezzeddine, Lara Abramian, Ali Koubeissi, Nikolay Vladimirov, Kamal H. Bouhadir

**Affiliations:** a Department of Natural Sciences, Lebanese American University, Beirut, Lebanon; b Department of Chemistry, American University of Beirut, Beirut, Lebanon; c SOLENIS LLC, Wilmington, DE, USA

**Keywords:** Polynucleotide analogues, cyclopolymerization, cyclo-copolymerization, pyrimidines, alkyldiallylammonium salts

## Abstract

We report the preparation and characterization of three pyrimidine-based monomers, specifically: 1-(2-diallylaminoethyl)uracil, 1-(2-diallylaminoethyl)thymine and 1-(2-diallylaminoethyl)cytosine. Monomer synthesis was initiated by reaction of the pyrimidine with ethylene carbonate to form the hydroxyethyl adduct which was subsequently chlorinated to afford the chloroethyl intermediate. Reaction of the chloroethyl derivatives with diallylamine resulted in the desired monomers. We demonstrated a two-fold increase in the overall yield of the three monomers in comparison to reported procedures. The cyclopolymerization and cyclo-copolymerization of 1-(2-diallylaminoethyl)pyrimidine trifluoroacetate salts in water resulted in low-yield homopolymers. In contrast, the neutral 1-(2-diallylaminoethyl)pyrimidines cyclo-copolymerized with sulfur dioxide and V-50 initiator to yield the corresponding copolymers in higher yields ranging from 30 to 60%.

## 1. Introduction

Facile synthetic protocols leading to functional modified oligodeoxynucleotides (ODNs) are valuable for their potential therapeutic and diagnostic applications [1–4]. A necessary prerequisite for such analogs is stability against biological enzymes that typically cleave the phosphodiester backbone in natural nucleic acids [5,6]. The literature is saturated with studies investigating structural modifications of the nucleic acid backbone, including the replacement of the phosphodiester linkage, the furanose cycle and/or the nucleobase. One convenient route to form homopolymers resembling modified ODNs is through the cyclopolymerization of nucleic base-substituted diallylamine derivatives. Although the cyclopolymerization of diallyl quaternary ammonium salts has been extensively studied [7,8], very little work has been reported on the cyclopolymerization of the alkyldiallylammonium derivatives [9–11]. Deprotonation of polymers prepared from alkyldiallylammonium salts will yield the corresponding neutral polymers which are likely to be soluble in organic solvents and expand their utility in a variety of applications.

We recently reported a facile route to homopolymers and copolymers via the polymerization of 1-(2-diallylaminoethyl)adenine, simultaneously replacing the phosphodiester and ribose moieties of natural ODNs with a hydrocarbon or sulfone backbone and a cyclopentane ring respectively [12]. Here, we have directed our attention to the related pyrimidine derivatives. Pyrimidines are heterocyclic aromatic compounds with a diazine nucleus that is prevalent in a diverse array of natural products. Pyrimidine derivatives have a multitude of medicinal properties and therapeutic applications and have been reported to possess anticancer [13], antimicrobial [14–16], antiviral [17], anti-inflammatory [18,19] and analgesic [20] activities. In this manuscript, we report the synthesis and polymerization of 1-(2-diallylaminoethyl)pyrimidines to yield a series of pyrimidine-substituted homopolymers and copolymers.

## 2. Experimental

### 2.1. Materials and methods

Reagents used in the syntheses were purchased from the Aldrich Chemical Company (Milwaukee, WI), ACROS Chemicals (Loughborough, United Kingdom), Fisher Scientific Company (Fair Lawn, NJ) and were used as received. Dioxane was dried over sodium metal and distilled directly before use. The water-soluble initiator 2,2’-azobis(2-methylpropionamidine)dihydrochloride (V-50) was obtained from Wako Pure Chemical Industries (Richmond, VA) and was used as received.

Melting points were determined on a Mettler Toledo FP62 apparatus and are uncorrected. NMR spectra were determined in deuterated solvents with tetramethylsilane (TMS) or sodium 2,2-dimethyl-2-silapentane-5-sulfonate (DSS) as the internal standards on a Bruker AV 300 NMR spectrometer. Chemical shifts are reported in ppm (δ) downfield relative to TMS or DSS. Infrared spectra were recorded as KBr pellets using a Nicolet 4700 FTIR spectrometer with a Hewlett Packard Desk jet 840C plotter. The IR bands are reported in wave numbers (cm^−1^). SEC analysis was performed on a liquid chromatograph consisting of a Waters Breeze solvent delivery system and Waters M717 autosampler (Waters Corporation, Milford, MA, USA), a DAWN EOS light scattering photometer and an OPTILAB rEX differential refractive index detector (Wyatt Technology Corporation, Santa Barbara, CA, USA). Aqueous oxalic acid (0.22 M) at 40 °C with nominal flow rate of 0.8 mL/min was used as the mobile phase. The separations were carried out on a PSS Novema pre-column connected in series to three PSS Novema columns (30, 1,000, 10,000 Å) from Polymer Standard Service (Amherst, MA, USA) (8.0 mm × 300 mm, 10 μm). All samples were prepared by stirring overnight in the mobile phase at a concentration of 1–2 mg/mL and filtered through 0.45 μm PVDF membrane filter.

#### 2.2. Experimental procedures

##### 2.2.1. 1-(2-Hydroxyethyl)uracil (**2a**)

A 500 mL round-bottom flask was charged with uracil (10 g, 90 mmol), anhydrous DMF (220 mL) and crushed sodium hydroxide pellets (0.66 g, 16.5 mmol). The mixture was heated for 10 min until the solution turned clear. Ethylene carbonate (9.88 g, 102 mmol) was added and the mixture was refluxed for 90 min and left to stir overnight at room temperature. The solvent was evaporated under reduced pressure, water (300 mL) was added to the residue and left to stand overnight. The mixture was filtered to remove unreacted uracil. Concentrated ammonia (1 drop) was added to the filtrate to adjust the pH to 11. The product was separated from the bis(hydroxyethyl)uracil byproduct with anion exchange chromatography (35 g, Dowex). The byproduct was eluted with aqueous ammonia (1 L, 10%). Compound **2a** was eluted with acetic acid (7 L, 0.1 M). The volume of the acidic fraction was reduced to 200 ml where unreacted uracil precipitated. The solid was removed and the filtrate was evaporated under reduced pressure to yield a solid that was recrystallized from dioxane, filtered and dried under reduced pressure to yield **2a** (4.2 g, 30%). m.p. 138–140 °C. ^1^H NMR (300 MHz, DMSO-d_6_) δ 3.55 (t, *J* = 5.3 Hz, 2H), 3.70 (t, *J* = 5.3 Hz, 2H), 4.70 (br s, 1H), 5.51 (d, *J* = 7.8 Hz, 1H), 7.54 (d, *J* = 7.8 Hz, 1H), 11.07 (br s, 1H); ^13^C NMR (75 MHz, DMSO-d_6_) δ 50.0, 58.4, 99.9, 146.6, 150.8, 163.8; FTIR 3447, 3015, 2963, 2881, 2806, 1691, 1626, 1468, 1423 cm^−1^. Spectroscopic data are consistent with those reported in the literature [5,6].

##### 2.2.2. 1-(2-Hydroxyethyl)thymine (**2b**)

A procedure similar to that of **2a** was followed to prepare **2b** (4.5 g, 33%). m.p. 161–162 °C. ^1^H NMR (300 MHz, DMSO-d_6_) δ 1.75 (s, 3H), 3.56 (t, *J* = 5.1 Hz, 2H), 3.68 (t, *J* = 5.2 Hz, 2H), 4.90 (br s, 1H), 7.43 (s, 1H), 11.20 (br s, 1H); ^13^C NMR (75 MHz, DMSO-d_6_) δ 49.8, 58.5, 107.8, 137.7, 151.5, 165.1; FTIR 3288, 3004, 2813, 1710, 1661, 1519, 1482, 1459, 1419, 1385, 1361, 1225, 1136, 1059, 1008, 927 cm^−1^. Spectroscopic data are consistent with those reported in the literature [21,22].

##### 2.2.3. 1-(2-Hydroxyethyl)cytosine (**2c**)

A 1 L round-bottom flask was charged with cytosine (10 g, 90 mmol), dry DMF (650 mL) and crushed sodium hydroxide pellets (1.8 g, 45 mmol). The suspension was stirred and heated until the solution was clear. Ethylene carbonate (8.95 g, 101.7 mmol) was added and the mixture was refluxed for twelve hours. The solvent was evaporated under reduced pressure and the solid was recrystallized from ethanol to yield **2c** (11.2 g, 80%). m.p. 228–230 °C. ^1^H NMR (300 MHz, DMSO-d_6_) δ 3.54 (t, *J* = 5.1 Hz, 2H), 3.67 (t, *J* = 5.4 Hz, 2H), 4.85 (br s, 1H), 5.61 (d, *J* = 7.2 Hz, 1H), 7.00 (br s, 2H), 7.48 (d, *J* = 6.9 Hz, 1H); ^13^C NMR (75 MHz, DMSO-d_6_) δ 51.2, 58.7, 92.5, 146.9, 155.8, 165.9; FTIR 3475, 3352, 3281, 2966, 2887, 1669, 1498 cm^−1^. Spectroscopic data are consistent with those reported in the literature [23].

##### 2.2.4. 1-(2-Chloroethyl)uracil (**3a**)

A 2 L round-bottom flask was charged with **2a** (14 g, 89.66 mmol), dry dioxane (450 mL) and freshly distilled pyridine (22.5 mL). A solution of freshly distilled thionyl chloride (31.74 g, 268 mmol) in dry dioxane (600 mL) was added drop-wise to the mixture. The mixture was refluxed for one hour and stirred overnight at room temperature. The solvent was evaporated under reduced pressure and the solid was recrystallized from ethanol to yield **3a** (14.35 g, 92%). m.p. 163–165 °C. ^1^H NMR (300 MHz, DMSO-d_6_) δ 3.85 (t, *J* = 5.8 Hz, 2H), 4.01 (t, *J* = 5.7 Hz, 2H), 5.59 (d, *J* = 7.9 Hz, 1H), 7.67 (d, *J* = 7.8 Hz, 1H), 11.35 (br s, 1H); ^13^C NMR (75 MHz, DMSO-d_6_) δ 42.1, 48.8, 100.5, 145.9, 150.7, 163.5; FTIR 3109, 3013, 2816, 1665, 1465, 1271, 1231, 904 cm^−1^. Spectroscopic data are consistent with those reported in the literature [5,6].

##### 2.2.5. 1-(2-Chloroethyl)thymine (**3b**)

A procedure similar to that of **3a** was followed to prepare **3b** (5.96 g, 97%). m.p. 203–205 °C. ^1^H NMR (300 MHz, DMSO-d_6_) δ 1.76 (s, 3H), 3.84 (t, *J* = 5.8 Hz, 2H), 3.97 (t, *J* = 5.8 Hz, 2H), 7.56 (s, 1H), 11.35 (br s, 1H); ^13^C NMR (75 MHz, DMSO-d_6_) δ 11.8, 42.1, 48.6, 108.2, 141.6, 150.8, 164.2; FTIR 3094, 2994, 2892, 2831, 1713, 1671, 1482, 907 cm^−1^. Spectroscopic data are consistent with those reported in the literature [21,22].

##### 2.2.6. 1-(2-Chloroethyl)cytosine (**3c**)

A procedure similar to that of **3a** was followed to prepare **3c** (1.1 g, 97%). m.p. 170–171 °C. ^1^H NMR (300 MHz, DMSO-d_6_) δ 3.89 (t, *J* = 5.7 Hz, 2H), 4.12 (t, *J* = 5.7 Hz, 2H), 6.09 (d, *J* = 7.8 Hz, 1H), 8.01 (d, *J* = 7.5 Hz, 1H); ^13^C NMR (75 MHz, DMSO-d_6_) δ 41.7, 50.1, 93.0, 147.5, 149.9, 159.8; FTIR 3314, 3109, 2868, 1758, 1713, 1674, 1536, 1178, 982 cm^−1^. Spectroscopic data are consistent with those reported in the literature [23].

##### 2.2.7. 1-(2-Diallylaminoethyl)uracil (**4a** from **3a**)

A 50 mL tube was charged with **3a** (0.5 g, 2.87 mmol), absolute ethanol (20 mL) and diallylamine (1.76 mL, 14.37 mmol). The tube was sealed and the mixture was refluxed at 90 °C for three days. The solvent was evaporated under reduced pressure to yield a solid residue that was dissolved in aqueous HCl (10 mL, 1 M). The aqueous solution was washed with CHCl_3_ (3 × 10 mL), basified with aqueous NaOH (10 mL, 1 M) and extracted with CHCl_3_ (3 × 10 mL). The organic layers were collected, dried over anhydrous MgSO_4_, filtered and the solvent was evaporated under reduced pressure to yield **4a** (0.65 g, 97%). m.p. 91–92 °C. ^1^H NMR (300 MHz, CDCl_3_) δ 2.63 (t, *J* = 7.8 Hz, 1H), 3.03 (d, *J* = 6.4 Hz, 4H), 3.71 (t, *J* = 5.8 Hz, 2H), 5.08 (m, 4H), 5.59 (d, *J* = 7.8 Hz, 1H), 5.66 (m, 2H), 7.23 (d, *J* = 7.8 Hz, 1H), 9.50 (br s, 2H); ^13^C NMR (75 MHz, CDCl_3_) δ 46.6, 51.1, 57.2, 100.9, 146.0, 151.1, 164.6; FTIR 3154, 3024, 2805, 1694, 1648, 1467, 1419, 1363, 1242, 920 cm^−1^.

##### 2.2.8. 1-(2-Diallylaminoethyl)thymine (**4b** from **3b**)

A procedure similar to that of **4a** was followed to prepare **4b** (1.23 g, 62%). m.p. 118 °C. ^1^H NMR (300 MHz, CDCl_3_) δ 1.91 (s, 3H), 2.71 (t, *J* = 5.9 Hz, 2H), 3.12 (d, *J* = 6.2 Hz, 4H), 3.76 (t, *J* = 5.9 Hz, 2H), 5.12 (m, 4H), 5.75 (m, 2H), 7.08 (s, 1H), 10.29 (br s, 1H); ^13^C NMR (75 MHz, CDCl_3_) δ 12.2, 46.6, 51.4, 57.2, 109.4, 117.9, 135.0, 141.8, 151.1, 164.8; FTIR 3163, 3072, 3024, 2811, 1700, 1653, 1485, 1423, 915 cm^−1^.

##### 2.2.9. 1-(2-Diallylaminoethyl)cytosine (**4c** from **3c**)

A procedure similar to that of **4a** was followed to prepare **4c** (0.68 g, 54%). ^1^H NMR (300 MHz, CDCl_3_) δ 2.65 (t, *J* = 5.6 Hz, 2H), 3.02 (d, *J* = 6.0 Hz, 2H), 3.70 (t, *J* = 5.6 Hz, 2H), 5.06 (m, 2H), 5.60 (m, 1H), 5.74 (d, *J* = 7.4 Hz, 1H), 7.24 (d, *J* = 7.1 Hz, 1H); ^13^C NMR (75 MHz, CDCl_3_) δ 48.1, 51.6, 57.2, 93.3, 117.8, 135.1, 146.9, 156.0, 165.2; FTIR 3352, 3139, 3005, 2976, 2924, 2799, 1656, 1614, 1523, 1486, 1456, 1383, 1269, 916, 808, 788, 678, 619 cm^−1^.

##### 2.2.10. ^3^ N-Benzoyluracil (**5a**)

A 1 L round-bottom flask was charged with uracil (25.17 g, 224.5 mmol), pyridine (130 mL) and acetonitrile (320 mL). Benzoyl chloride (110 mL, 0.938 mol) was added in one portion and the mixture was stirred for four days at room temperature. As the reaction progressed, the solution became cloudy and then turned to clear orange. The solvent was removed under reduced pressure and the viscous slurry was dissolved in CH_2_Cl_2_ (800 mL). The resulting solution was washed with saturated aqueous NaHCO_3_ (6 × 100 mL) and then with water (800 mL), dried over anhydrous Na_2_SO_4_ and filtered. The solvent was evaporated under reduced pressure to yield ^1^ *N*,^3^ *N*-dibenzoyluracil as a yellowish solid. Dioxane (500 mL) and aqueous K_2_CO_3_ (500 mL, 0.5 M) were added to the crude solid and stirred for two days at room temperature during which all the solid dissolved. The resulting solution was acidified with concentrated HCl and water (30 mL) was added to precipitate the product. The precipitate was filtered, washed with water and dried to yield **5a** as a white solid (35.34 g, 75%). m.p. 181–182 °C. ^1^H NMR (300 MHz, DMSO-d_6_) δ 5.73 (d, *J* = 7.7 Hz, 1H), 7.60 (t, *J* = 7.8 Hz, 2H), 7.66 (d, *J* = 7.7 Hz, 1H), 7.76 (t, *J* = 7.4 Hz, 1H), 7.94 (d, *J* = 8.5 Hz, 2H); ^13^C NMR (75 MHz, DMSO-d_6_) δ 99.9, 129.3, 130.1, 131.2, 135.3, 143.3, 150.0, 162.8, 169.9; FTIR 3324, 3220, 3116, 2967, 1747, 1653, 1230, 934 cm^−1^. Spectroscopic data are consistent with those reported in the literature [24,25].

##### 2.2.11. ^3^ N-Benzoylthymine (**5b**))

A 1 L round-bottom flask was charged with thymine (12.61 g, 100 mmol), pyridine (60 mL), and acetonitrile (150 mL). Benzoyl chloride (23.5 mL, 200 mmol) was added in one portion and the mixture was stirred for four days at room temperature. As the reaction progressed, the solution became cloudy and then turned to clear orange. The solvent was evaporated under reduced pressure and the viscous slurry was dissolved in CH_2_Cl_2_ (500 mL). The resulting solution was washed with saturated aqueous NaHCO_3_ (100 mL), dried over anhydrous MgSO_4_ and filtered. The solvent was evaporated under reduced pressure to yield ^1^ *N*,^3^ *N*-dibenzoylthymine as a yellowish solid. The crude solid was dissolved in dioxane (200 mL) and aqueous K_2_CO_3_ (200 mL, 0.5 M). The solution was stirred for 45 min at room temperature, filtered to remove insoluble particles. The filtrate was acidified with concentrated HCl where a white solid precipitated. The solid was filtered, washed with water and dried under reduced pressure to yield **5b** (18.9 g, 82%). m.p. 177–178 °C. ^1^H NMR (300 MHz, DMSO-d_6_) δ 1.82 (s, 3H), 7.53 (s, 1H), 7.58 (t, *J* = 7.7 Hz, 2H), 7.76 (t, *J* = 7.6 Hz, 1H), 7.93 (d, *J* = 7.8 Hz, 2H), 11.4 (br s, 1H); ^13^C NMR (75 MHz, DMSO-d_6_) δ 11.6, 107.8, 129.3, 130.1, 131.3, 135.2, 138.7, 149.9, 163.5, 170.1; FTIR 3212, 3090, 2955, 1750, 1704, 1638, 1596, 1451, 1423, 1222, 966 cm^−1^. Spectroscopic data are consistent with those reported in the literature [24,25].

##### 2.2.12. 1-(2-Diallylaminoethyl)uracil (**4a** from **5a**)

A 250 mL three-neck round-bottom flask was charged with ^3^ *N*-benzoyluracil **5a** (2 g, 9.25 mmol), bromoethanol (1.48 g, 11.1 mmol), PPh_3_ (2.45 g, 9.25 mmol) and dry dioxane (60 mL). The flask was partially immersed in an ice-water bath and a solution of DIAD (0.985 g, 9.25 mmol) in dry dioxane (60 mL) was added drop-wise under an atmosphere of nitrogen. The solution turned clear yellow halfway through the addition. The ice-water bath was removed and the solution was stirred for one day at room temperature under an atmosphere of nitrogen. The solution was transferred to a 200 mL round-bottom flask, charged with diallylamine (2.77 g, 27.75 mmol) and refluxed for two days. The solvent was evaporated under reduced pressure and the residue was acidified with aqueous HCl (50 mL, 10%) and washed with CH_2_Cl_2_ (3 × 25 mL). The aqueous solution was then neutralized by adding aqueous NaOH (50 mL, 10%) and extracted with CH_2_Cl_2_ (3 × 50 mL). The organic layers were combined, dried over anhydrous Na_2_SO_4_ and filtered. The solvent was evaporated under reduced pressure to yield **4a** (1.2 g, 55%).

##### 2.2.13. 1-(2-Diallylaminoethyl)thymine (**4b** from **5b**)

A 100 mL two-neck round-bottom flask was charged with ^3^ *N*-benzoylthymine **5b** (0.5 g, 2.17 mmol), bromoethanol (0.33 g, 2.6 mmol), PPh_3_ (1.149 g, 4.34 mmol) and dry dioxane (20 mL). The flask was partially immersed in an ice-water bath and a solution of DIAD (0.93 g, 0.93 mmol) in dry dioxane (10 mL) was added drop-wise under an atmosphere of nitrogen. The solution turned clear halfway through the addition. The ice-water bath was removed and the solution was stirred for four days at room temperature under an atmosphere of nitrogen. The solution was transferred to a 100 mL round-bottom flask, charged with diallylamine (0.54 g, 5.42 mmol) and refluxed for three days. The solvent was evaporated under reduced pressure and the residue was acidified with aqueous HCl (50 mL, 10%) and washed with CH_2_Cl_2_ (3 × 25 mL). The aqueous solution was then neutralized by adding aqueous NaOH (50 mL, 10%) and extracted with CH_2_Cl_2_ (3 × 50 mL). The organic layers were combined, dried over anhydrous Na_2_SO_4_ and filtered. The solvent was evaporated under reduced pressure to yield **4b** (0.25 g, 46%).

##### 2.2.14. N^4^-Isobutyrylcytosine (**5c**)

A 50 mL round-bottom flask was charged with dry cytosine (1 g, 9 mmol), isobutyric anhydride (4.7 g, 29.71 mmol), and dry DMF (25 mL). The mixture was refluxed for two hours and left to stand overnight. The solid precipitate was filtered, washed with water:ethanol (3 mL, 1:1) and dried to yield 1.2 g of **5c** as white crystals. The yellow filtrate was evaporated to dryness and an additional 0.1 g of the product was recrystallized (80%). m.p. decomposes above 300 °C. ^1^H NMR (300 MHz, DMSO-d_6_) δ 1.06 (d, *J* = 6.8 Hz, 6H), 2.72 (m, 1H), 7.16 (d, *J* = 7.0 Hz, 1H), 7.83 (d, *J* = 7.0 Hz, 1H), 11.10 (br s, 1H); ^13^C NMR (75 MHz, DMSO-d_6_) δ 18.9, 34.7, 94.5, 147.1, 156.2, 163.4, 177.6; FTIR 3215, 3156, 2985, 2604, 2254, 2003, 1936, 1866, 1711, 1699, 1621, 1471, 1452, 1419, 1385, 1343, 1306, 1229, 1181, 1141, 1101 cm^−1^. Spectroscopic data are consistent with those reported in the literature [26].

##### 2.2.15. N^4^-Isobutyryl-1-(2-diallylaminoethyl)cytosine(**7c**)

A 250 mL three-neck round-bottom flask was charged with *N* ^*4*^-isobutyrylcytosine (2 g, 11.038 mmol), bromoethanol (1.7 g, 13.2 mmol), PPh_3_ (5.84 g, 22.07 mmol), and dry dioxane (160 mL). The flask was partially immersed in an ice-water bath and a solution of DIAD (4.68 g, 22.076 mmol) in dry dioxane (60 mL) was added drop-wise under an atmosphere of nitrogen. The ice-water bath was removed and the solution was stirred for one day at room temperature. The solution was transferred to a 500 mL round-bottom flask, charged with diallylamine (2.2 g, 22 mmol) and refluxed for five days. The solvent was evaporated under reduced pressure and the residue was acidified with aqueous HCl (50 mL, 10%), washed with CHCl_3_ (3 × 30 mL), neutralized with aqueous NaOH (50 mL, 10%) and the product was extracted with CHCl_3_ (3 × 50 mL). The organic layers were collected, dried over anhydrous Na_2_SO_4_ and filtered. The solvent was evaporated under reduced pressure to yield an oily residue that was triturated with hexane. The pale brown solid was filtered and dried under reduced pressure to yield **7c** (1.23 g, 36.6%). ^1^H NMR (300 MHz, CDCl_3_) δ 1.28 (d, *J* = 6.8 Hz, 6H), 2.60 (m, 1H), 2.78 (d, *J* = 5.2 Hz, 2H), 3.09 (d, *J* = 6.1 Hz, 4H), 3.89 (t, *J* = 5.1 Hz, 2H), 5.13 (m, 4H), 5.67 (m, 2H), 7.34 (d, *J* = 7.0 Hz, 1H), 7.62 (d, *J* = 7.1 Hz, 1H), 8.34 (br s, 1H); ^13^C NMR (75 MHz, CDCl_3_) δ 19.1, 36.4, 48.8, 51.1, 57.1, 95.5, 118.0, 134.8, 150.0, 156.0, 162.4, 177.4; FTIR 3181, 3076, 2976, 2931, 2815, 1712, 1654, 1555, 1491, 1467, 1426, 1361, 1311, 1210, 919 cm^−1^.

##### 2.2.16. 1-(2-Diallylaminoethyl)cytosine (**4c** from **7c**)

A 100 mL three-neck round-bottom flask immersed in an ice-water bath was charged with methanol (12 mL) and sodium (0.124 g, 5.4 mmol). A solution of **7c** (0.5 g, 1.64 mmol) and methanol (10 mL) was added drop-wise under an atmosphere of nitrogen. The ice-water bath was removed and the solution was refluxed for 3.5 h and left to stir overnight at room temperature. The flask was immersed in an ice-water bath and water (17 mL) was added to the mixture. The solution was concentrated to 1/10 its initial volume under reduced pressure. The solution was acidified with aqueous HCl (5 mL, 10%) and washed with CHCl_3_ (3 × 25 mL). The aqueous layer was then neutralized with aqueous NaOH (5 mL, 10%) and extracted with CHCl_3_ (4 × 25 mL). The organic layers were collected, dried over anhydrous Na_2_SO_4_ and filtered. The solvent was evaporated under reduced pressure to yield **4c** (0.36 g, 85%).

#### 2.3. Procedure for the cyclopolymerization of **4a**

A 25 mL tube was charged with **4a** (0.216 g, 0.92 mmol), aqueous TFA (0.934 mL, 3.473 M) and V-50 (5.0 mg, 0.018 mmol). The tube was sealed with a septum and the solution was purged with nitrogen gas for 10 min. The mixture was stirred and heated at 70 °C for 48 h. The product was precipitated in ethanol, filtered and dried at 80 °C under reduced pressure to yield **8a** (49 mg, 23%). ^1^H NMR (300 MHz, D_2_O) δ 1.32 (s, br), 2.48 (s, br), 3.13 (s, br), 3.60 (s, br), 4.21 (s, br), 5.88 (d, *J* = 7.2 Hz, 1H), 7.67 (d, *J* = 6.6 Hz, 1H); ^13^C NMR (75 MHz, D_2_O) δ 27.9, 42.1, 47.0, 56.9, 60.5, 105.0, 148.8, 155.1, 165.7; FTIR 3726, 3702, 3625, 3598, 1679 cm^−1^.

#### 2.4. Procedure for the cyclopolymerization of **4b**

A 50 mL tube was charged with **4b** (0.459 g, 0.92 mmol), aqueous TFA (1.05 mL, 3.473 M) and V-50 (10.0 mg, 0.036 mmol). The tube was sealed with a septum and the solution was purged with nitrogen gas for 10 min. The mixture was stirred and heated at 70 °C for 48 h. The product was precipitated in ethanol, filtered and dried at 80 °C under reduced pressure to yield **8b** (30 mg, 7%). ^1^H NMR (300 MHz, D_2_O) δ 1.36 (s, br), 1.88 (s, 3H), 2.53 (s, br), 3.34 (s, br), 3.61 (s, br), 4.18 (s, br), 7.53 (s, 1H); ^13^C NMR (75 MHz, D_2_O) δ 14.0, 27.4, 29.1, 43.2, 46.9, 57.1, 61.1, 114.3, 144.6, 155.2, 169.2; FTIR 3726, 3703, 3628, 3599, 1668 cm^−1^.

#### 2.5. Procedure for the cyclopolymerization of **4c**

A 25 mL tube was charged with **4c** (0.345 g, 1.47 mmol), aqueous TFA (0.758 mL, 3.473 M) and V-50 (20 mg, 0.0737 mmol). The tube was sealed with a septum and the solution was purged with nitrogen gas for 10 min. The mixture was stirred and heated at 70 °C for 48 h. The product was precipitated in ethanol to yield traces of **8c**.

#### 2.6. Procedure for the cyclo-copolymerization of **4a**

A 25 mL tube was charged with **4a** (0.217 g, 0.92 mmol), a solution of SO_2_ (0.11 g, 1.7 mmol) in MeOH (0.328 mL) and V-50 (5 mg, 0.018 mmol). The solution was freeze-thaw-degassed (three cycles) and sealed. The mixture was heated at 70 °C for 48 h with stirring. The mixture was dissolved in TFA (3 mL), sonicated for 15 min, stirred for 5 min, precipitated in methanol (8 mL), filtered and dried at 80 °C under reduced pressure to yield **9a** (99 mg, 46%). ^1^H NMR (300 MHz, D_2_O) δ 3.01 (s, br), 3.35 (s, br), 3.39 (s, br), 3.71 (s, br), 4.24 (s, br), 5.88 (d, *J* = 7.5 Hz, 1H), 7.67 (d, *J* = 7.5 Hz, 1H); ^13^C NMR (75 MHz, D_2_O) δ 35.8, 37.9, 46.8, 53.1, 56.9, 59.4, 105.1, 148.7, 155.5, 168.8; FTIR 1682, 1461, 1252, 1199, 1129 cm^−1^.

#### 2.7. Procedure of the cyclo-copolymerization of **4b**

A 25 mL tube was charged with **4b** (0.229 g, 0.92 mmol), a solution of SO_2_ (0.16 g, 2.5 mmol) in MeOH (0.562 mL) and V-50 (5 mg, 0.018 mmol). The solution was freeze-thaw-degassed (three cycles) and sealed. The mixture was heated at 70 °C for 48 h with stirring. The mixture was dissolved in water (3 mL) with 1 drop of TFA, sonicated for 15 min, stirred for 5 min, precipitated in methanol (8 mL), filtered and dried at 80 °C under reduced pressure to yield **9b** (0.17 g, 60%). ^1^H NMR (300 MHz, D_2_O) δ 1.90 (s, 3H), 3.12 (s, br), 3.42 (s, br), 3.73 (s, br), 4.00 (s, br), 4.22 (s, br), 7.34 (s, 1H); ^13^C NMR (75 MHz, D_2_O) δ, 14.0, 37.0, 39.2, 47.7, 54.8, 57.6, 60.6, 115.7, 144.4, 155.7, 169.1; FTIR 1679, 1473, 1302, 1257, 1222, 1125, 1037 cm^−1^.

#### 2.8. Procedure for the cyclo-copolymerization of **4c**

A 25 mL tube was charged with **4c** (0.431 g, 1.84 mmol), a solution of SO_2_ (0.35 g, 5.5 mmol) in MeOH (0.99 mL) and V-50 (5 mg, 0.036 mmol). The solution was freeze-thaw-degassed (three cycles) and sealed. The mixture was heated at 70 °C for 48 h with stirring. The yellowish precipitate was dissolved in water (3 mL), sonicated for 15 min, stirred for 5 min, precipitated in methanol, filtered and dried at 80 °C under reduced pressure to yield **9c** (130 mg, 30%). ^1^H NMR (300 MHz, D_2_O) δ 3.05 (s, br), 3.40 (s, br), 3.60 (s, br), 3.74 (s, br), 4.31 (s, br), 6.22 (d, *J* = 7.2 Hz, 1H), 7.88 (d, *J* = 7.5 Hz, 1H); ^13^C NMR (75 MHz, D_2_O) δ 36.1, 38.1, 48.1, 52.1, 57.3, 59.8, 98.4, 151.7, 151.9, 162.5; FTIR 3727, 3703, 3626, 3599, 1682, 1200, 668, 649 cm^−1^.

#### 2.9. Procedure for the cyclo-copolymerization of **4a** (under acidic conditions)

A 25 mL tube was charged with **4a** (0.506 g, 2.15 mmol), a solution of SO_2_ (0.048 g, 1.5 mmol) in water (1 mL), TFA (200 μL, 2.6 mmol) and V-50 (10 mg, 0.036 mmol). The solution was freeze-thaw-degassed (three cycles) and sealed. The mixture was heated at 70 °C for 48 h with stirring. The mixture was dissolved in water (5 mL) and precipitated in methanol (20 mL), filtered and dried at 80 °C under reduced pressure to yield **9a** (59 mg, 9%).

#### 2.10. Procedure of the cyclo-copolymerization of **4b** (under acidic conditions)

A 25 mL tube was charged with **4b** (0.244 g, 0.98 mmol), a solution of SO_2_ (24 mg, 0.75 mmol) in water (0.5 mL), TFA (100 μL, 1.3 mmol) and V-50 (5 mg, 0.018 mmol). The solution was freeze-thaw-degassed (three cycles) and sealed. The mixture was heated at 70 °C for 48 h with stirring. The mixture was dissolved in water (3 mL) and precipitated in methanol (10 mL), filtered and dried at 80 °C under reduced pressure to yield **9b** (84 mg, 27%).

#### 2.11. Procedure for the cyclo-copolymerization of **4c** (under acidic conditions)

A 25 mL tube was charged with **4c** (0.512 g, 2.184 mmol), a solution of SO_2_ (48 mg, 1.5 mmol) in water (1 mL), TFA (200 μL, 2.6 mmol) and V-50 (12 mg, 0.864 mmol). The solution was freeze-thaw-degassed (three cycles) and sealed. The mixture was heated at 70 °C for 48 h with stirring. The mixture was dissolved in water (5 mL) and precipitated in methanol (20 mL), filtered and dried at 80 °C under reduced pressure to yield **9c** (109 mg, 15%).

## 3. Results and discussion

**Scheme 1.**
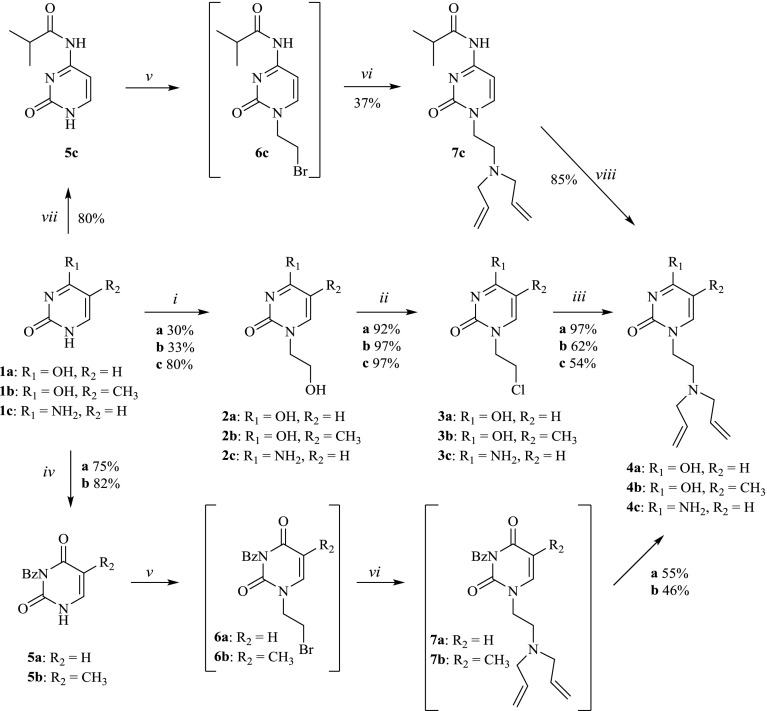
Synthesis of compounds **4a, 4b & 4c**. Reagents and Conditions: (i) Ethylene carbonate, NaOH, DMF, reflux, 24 h; (ii) SOCl_2_, dry pyridine, dry dioxane, reflux, 2 h; (iii) diallylamine, absolute EtOH, reflux, 3 d; (iv) a. Benzoyl chloride, pyridine, acetonitrile, r.t., 4 d b. K_2_CO_3_, dioxane; (v) DIAD, Ph_3_P, BrCH_2_CH_2_OH, dry dioxane, r.t., 24 h; (vi) diallylamine, dry dioxane, reflux, 2–5 d; (vii) Isobutyric anhydride, dry DMF, reflux, 2 h; (viii) NaOMe, MeOH, reflux, 3.5 h then r.t., 24 h.

The preparation of the pyrimidine monomers (Scheme 1) was initiated by heating uracil **1a**, thymine **1b** or cytosine **1c** with ethylene carbonate in dry DMF to afford the corresponding 1-(2-hydroxyethyl)pyrimidine **2a–2c** that was subsequently chlorinated with freshly distilled thionyl chloride to yield 1-(2-chloroethyl)pyrimidine **3a–3c** [21–23,26]. Refluxing compounds **3a–3c** with excess diallylamine in ethanol formed compounds **4a–4c**. The overall yields of compounds **4a–4c** from the corresponding pyrimidine were 28% for the uracil adduct **4a**, 20% for the thymine adduct **4b** and 42% for the cytosine adduct **4c**.

The relatively low overall yields of the uracil and thymine adducts in comparison to the cytosine derivative are related to the hydroxyethylation step that resulted in two isomers and entailed a time-consuming separation step resulting in low yields of **2a** and **2b**. This necessitated an alternative synthetic pathway utilizing the Mitsunobu reaction [23,27–30] to couple the protected pyrimidine bases **5a**-**5c** with 2-bromoethanol to form compounds **6a**-**6c**. The *N*-1 and *N*-3 positions of uracil and thymine were protected with benzoyl chloride followed by selective hydrolysis at the *N*-1 position using reported procedures [24,25] to afford compounds **5a** and **5b.** The monoprotected pyrimidine derivatives **5a** and **5b** were subsequently coupled to bromoethanol via the Mitsunobu reaction with triphenylphosphine (Ph_3_P) and diisopropylazodicarboxylate (DIAD) to yield **6a** and **6b** (not isolated). Refluxing **6a** and **6b** with excess diallylamine in dry dioxane afforded **7a** and **7b** (not isolated) that were subsequently deprotected to yield 1-(2-diallylaminoethyl)uracil **4a** and 1-(2-diallylaminoethyl)thymine **4b** in 41% and 38% overall yield from uracil and thymine respectively. In a similar procedure, the *N*-3 position of cytosine was protected with isobutyric anhydride to form **5c** that was afterwards coupled to bromoethanol to yield **6c** (not isolated). Refluxing **6c** with excess diallylamine in dry dioxane afforded **7c** [31]. The hydrolysis of the isobutyryl group was accomplished with sodium methoxide to form **4c** in 25% overall yield from cytosine. As seen above, the overall yields of both the uracil and thymine adducts were significantly increased via the Mitsunobu pathway in contrast to the cytosine derivative.

**Scheme 2.**
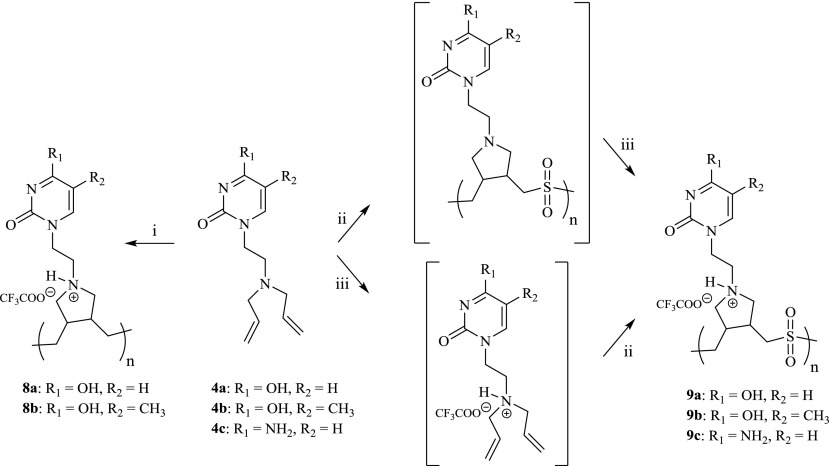
Cyclopolymerization and cyclo-copolymerization of compounds **4a–4c**. Reagents and Conditions: (i) TFA/H_2_O, V-50, 70 °C, 48 h; (ii) SO_2_/MeOH, V-50, 70 °C, 48 h; (iii) TFA/H_2_O.

Our interest in cyclopolymerization reactions of diallyl monomers was triggered from work reported by Butler and co-workers [32]. The polymerization of diallylamine and various *N*-substituted diallylamines is well documented to yield polymers with almost exclusively five-membered rings [33–35]. Our group has previously investigated the polymerization of a functionalized adenine monomer, and we expanded the project to study polymerization reactions of the pyrimidine derivatives **4a–4c**. The cyclopolymerization of compounds **4a**-**4c** (Scheme 2) was initially attempted under acidic conditions in water and initiated with 2,2’-azobis(2-methylpropionamidine)dihydrochloride (V-50) at 70 °C. While the uracil adduct **4a** formed polymer **8a** in a moderate yield of 23%, thymine derivative **4b** produced only 7% of the expected polymer **8b** (Entries 1 and 2, Table 1). In comparison, the functionalized cytosine **4c** failed to react under these conditions with only traces of the polymer observed (Entry 3, Table 1). Attempts to cyclopolymerize 1-(2-diallylaminoethyl)pyrimidines **4a–c** under neutral conditions in methanol failed and lead to recovery of the starting monomers (Entries 4–6, Table 1).

**Table 1.** Reaction conditions for the cyclopolymerization and cyclo-copolymerization of **4a–4c**.^a^

Entry	Compound	Solvent	Comonomer	TFA	Yield (%)^b^	M_n_ × 10^3^ (g/mol)^c^	M_w_ × 10^3^ (g/mol)^c^	M_z_ × 10^3^ (g/mol)^c^	PDI^d^	DP^e^
1	**4a**	H_2_O	–	1eq.	23	23.8	23.8	23.9	1.0	68
2	**4b**	H_2_O	–	1eq.	7	–	–	–	–	–
3	**4c**	H_2_O	–	1eq.	trace	–	–	–	–	–
4	**4a**	MeOH	–	–	NR	–	–	–	–	–
5	**4b**	MeOH	–	–	NR	–	–	–	–	–
6	**4c**	MeOH	–	–	NR	–	–	–	–	–
7	**4a**	H_2_O	SO_2_	1eq.	9	27.0	47.0	72.9	1.7	135
8	**4b**	H_2_O	SO_2_	1eq.	27	8.47	9.96	11.7	1.2	27
9	**4c**	H_2_O	SO_2_	1eq.	15	11.5	13.0	14.8	1.1	37
10	**4a**	MeOH	SO_2_	–	46	4.34	4.35	4.36	1.0	12
11	**4b**	MeOH	SO_2_	–	60	3.16	3.38	3.66	1.1	9
12	**4c**	MeOH	SO_2_	–	30	11.7	12.8	14.1	1.1	37

a All reactions were conducted in sealed tubes after degassing with nitrogen gas for 10 min (for aqueous solutions) or freeze-thawed-degassed (3 cycles) for methanol. Reactions were initiated with 2,2’-azobis(2-methylpropionamidine) dihydrochloride (V-50) with an initiator:monomer ratio of 1:50 (for **4a)**, 1:25 **(**for **4b)** and 1:20 **(**for **4c)** and heated at 70 °C for 48 h.

b Isolated yield after precipitation in ethanol.

c The separations were carried out on a PSS Novema pre-column connected in series to three PSS Novema columns (30, 1000, 10,000 Å). Aqueous oxalic acid (0.22 M) was used as the mobile phase at 40 °C with nominal flow rate of 0.8 mL/min.

d PDI = polydispersity index (M_w_/M_n_).

e DP = degree of polymerization.

We investigated the cyclo-copolymerization of the nucleobase olefinic derivatives **4a**-**4c** with sulfur dioxide which reportedly increases solubility and flexibility in the propagating chain and thus leads to high molecular weight polymers [36]. The cyclo-copolymerization reactions of the nucleobase derivatives **4a**-**4c** with an aqueous sulfur dioxide solution were initially evaluated in the presence of trifluoroacetic acid, which converts the reacting monomers to their corresponding ammonium salts. Cyclo-copolymerization of **4a** under acidic conditions resulted in 9% yield of polymer **9a** whereas polymer **9b** was isolated in 27% yield (from **4b**) and **4c** polymerized to **9c** in 15% yield (Entries 7–9, Table 1).

Interestingly, cyclo-copolymerizations of the neutral diallyl nucleobase derivatives **4a–c** with SO_2_, followed by protonation with TFA, resulted in formation of the corresponding copolymers in relatively higher yields. Attempts to manipulate conditions to increase the yield included changing initial concentrations of the monomer and initiator and ultimately we found each reaction had different initiator:monomer ratios for optimal polymer yields. For example, the uracil-derived olefin **4a** reacted with SO_2_ to form polymer **9a** in 46% yield when initiated in a 1:50 V-50:monomer ratio at 70 °C (Entry 10, Table 1). Decreasing the ratio led to significant reductions of polymer yields. These results are consistent with those obtained for the cyclo-copolymerization of 9-(2-diallylaminoethyl)adenine [12]. In comparison, **4b** and **4c** cyclo-copolymerized with SO_2_ under similar conditions to yield polymers **9b** and **9c** in 60% and 30% yield (Entries 11,12, Table 1) using initiatior:monomer ratios of 1:25 and 1:20 respectively.

**Figure 1.**
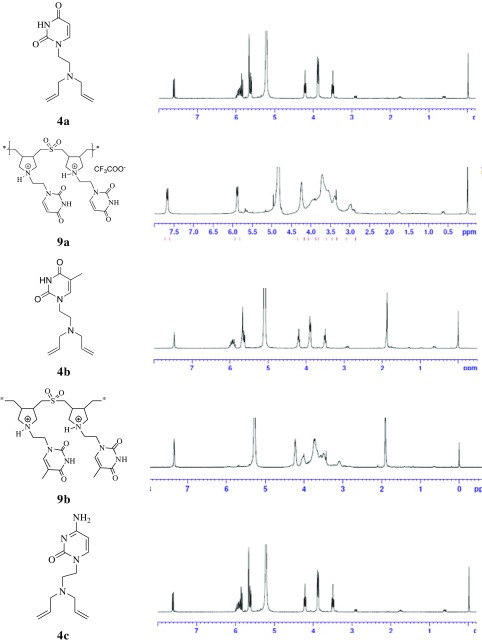
^1^H NMR spectra of monomers **4a–c** and pyrimidine-SO_2_-copolymers **9a–c**.

Importantly, formation of the polymers was supported by NMR spectroscopic data. The ^1^H NMR spectra of the precipitated products do not display the characteristic signals of vinylic hydrogens at δ 5.1, 5.6 and 5.7 of the starting monomers. The appearance of new signals in the ^1^H NMR spectra between ᵟ 3.05 and 4.31 for each product is indicative of formation of a pyrrolidine ring structure (Figure 1).

In addition, the absence of characteristic terminal vinylic ^13^C NMR signals and the appearance of new aliphatic signals in the precipitated products can be attributed to the formation of the expected cyclopolymers. For instance, the ^13^C NMR spectrum of the uracil-based cyclo-copolymerization product **9a** did not display any olefinic peaks but showed new signals at ᵟ 38.0 and 59.4 corresponding to the newly formed pyrrolidine ring system. Similar changes were observed for the thymine and cytosine-based molecules as well.

**Scheme 3.**
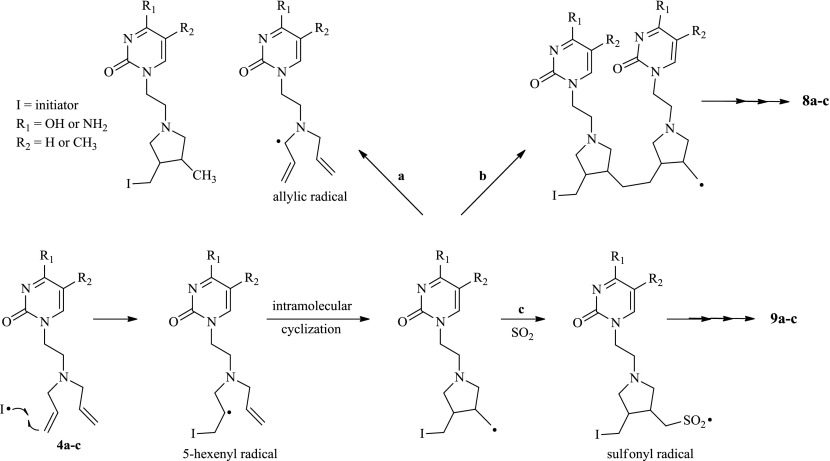
Mechanism for the cyclo-copolymerization of 1-(2-diallylaminoethyl)-pyrimidines.

The proposed mechanism for the cyclo-copolymerization reactions of the neutral 1-(2-diallylaminoethyl)pyrimidines is depicted in Scheme 3. The initiating radical attacks the terminal olefin of the alkyldiallylamine forming the 5-hexenyl radical that cyclizes via the 5-exo-trig mode to yield a highly reactive and nucleophilic primary radical. This radical could either abstract an allylic hydrogen from another monomer (degradative chain transfer) to yield a stable allylic radical (Pathway a) or attack another monomer (intermolecular propagation Pathway b). However, neutral diallyl monomers are known to have effective degradative chain transfer reactions [37,38] and this explains why the cyclopolymerization of compounds **4a–c** failed under the conditions investigated in this study.

Copolymers of olefins and SO_2_ are generally obtained in good yields [39–41]. The polymerization efficiency is ascribed to the formation of a complex between the diallyl groups and SO_2_ that facilitates the addition of the primary radical to SO_2_ forming a stable sulfonyl radical (Pathway c) that attacks another monomer to yield a propagating polymer chain. It is assumed that the flexibility of the sulfonyl radical introduced into the propagating polymer reduces the rigidity and increases the solubility of the propagating chain resulting in increased polymer yields [36]. This is consistent with the results obtained from the copolymerization of **4a–c** with SO_2_ in the absence of TFA with isolated yields ranging from 30% for the cytosine monomer to 60% for the thymine monomer.

## 4. Conclusions

We have reported the synthesis of three 1-(2-diallylaminoethyl)pyrimidines from the corresponding pyrimidine bases following two synthetic protocols. The overall yields of the uracil and thymine adducts were higher following the Mitsunobu pathway, whereas the cytosine adduct was prepared in higher yield via the hydroxyethylation route. The free-radical cyclopolymerization and cyclo-copolymerization of the three derivatives exhibited low degree of polymerization under acidic conditions. In contrast, the cyclo-coplymerization of the neutral 1-(2-diallylaminoethyl) pyrimidines with SO_2_ resulted in relatively higher yields.

## Disclosure statement

No potential conflict of interest was reported by the authors.

## Funding

This work was supported by the University Research Board (URB) at the American University of Beirut, the Lebanese National Council for Scientific Research (LNCSR), the Fulbright Scholar Program, the Arab Fund Fellowships Program, the Shair CRSL Research Fund and the Lebanese American University School Research and Development Council.
